# Type D Personality, Stress Level, Life Satisfaction, and Alcohol Dependence in Older Men

**DOI:** 10.3389/fpsyt.2021.712508

**Published:** 2021-10-11

**Authors:** Grzegorz Bejda, Agnieszka Kułak-Bejda, Napoleon Waszkiewicz, Elzbieta Krajewska-Kułak

**Affiliations:** ^1^The School of Medical Science in Białystok, Białystok, Poland; ^2^Department of Psychiatry, Medical University of Białystok, Białystok, Poland; ^3^Department of Integrated Medical Care, Medical University of Białystok, Białystok, Poland

**Keywords:** elderly, alcohol dependence, stress, life satisfaction, personality

## Abstract

Alcohol consumption among older adults is becoming an increasing public health problem due to the rapidly growing elderly population. There is a theory that Type D personality is positively correlated with alcohol dependence. The study aimed to assess the style of coping with stress, emotions and anxiety in elder men addicted to alcohol and the relationship between the above. The study included 170 men aged 60 years and older (mean age - 63 ± 3.1 years) addicted to alcohol staying in the Department of Alcohol Addiction Therapy for Men. They were tested with the questionnaire sheet and the following scales: Perceived Family Wealth (PFW), Family Affluence Scale (FAS), Cantril's Ladder of Life Scale, Satisfaction with Life Scale, Type D Personality Scale-14 (DS14), and the 10-item Perceived Stress Scale (PSS-10). The respondents' wealth on a scale of 1–5 points was assessed on avg. 3.1 ± 0.2. The above was confirmed by the results of the FAS scale study, where the respondents obtained an average of 3.9 ± 1.9 (min. 1, max. 8), which proves their average level of affluence. The evaluation of the satisfaction with life using Cantril's Ladder showed that the respondents were also satisfied with life on average (on average 5.5 ± 1.9). The assessment of life satisfaction using the Satisfaction with Life Scale (SWLS) scale allowed for the conclusion that the respondents were very dissatisfied with their lives (mean 17.2 ± 4.9). The evaluation of the measurement of perceived stress (PSS-10 scale) showed that the respondents obtained an average of 23.5 ± 3.7, and on the sten scale, a mean of 7.7 ± 0.98, which proves a high level of perceived stress. The study using the DS14 scale showed that the respondents were in the negative emotionality (NE) subscale - 17.4 ± 4.5 points, and in the HS scale - 16.2 ± 3.2, which proves that they can be classified as a Type D personality. The participants were very dissatisfied with their lives, with a high perceived stress and Type D personality.

## Introduction

Alcoholism, as a multidimensional phenomenon, can be considered in interdisciplinary categories, i.e., on the physiological, medical, psychiatric, psychological and social levels, due to both its specific conditions and specific consequences.

It should be emphasized that alcohol addiction becomes a social and medical problem in the case of older adults (1). The risk of developing alcohol problems does not decrease with age. Unfortunately, there are cases of medical misdiagnosis that attribute the effects of alcohol to the aging process, e.g., cognitive impairment, malnutrition and unsteady gait (1).

Alcohol use disorder (AUD) has been associated with neurodegenerative diseases such as Alzheimer's and Parkinson's disease (2). Prolonged excessive alcohol intake contributes to increased production of reactive oxygen species that trigger neuroimmune response and cellular apoptosis and necrosis *via* lipid peroxidation, mitochondrial, protein and DNA damage. In addition, long-term binge alcohol consumption upregulates the glutamate receptors, glucocorticoids. It reduces the reuptake of glutamate in the central nervous system, resulting in glutamate excitotoxicity and eventually mitochondrial injury and cell death.

In this review, we delineate the following principles in alcohol-induced neurodegeneration: (1) alcohol-induced oxidative stress; (2) neuroimmune response toward increased oxidants and lipopolysaccharide; (3) glutamate excitotoxicity and cell injury; (4) interplay between oxidative stress, neuroimmune response and excitotoxicity leading to neurodegeneration; and (5) potential chronic alcohol intake-induced development of neurodegenerative diseases, including Alzheimer's and Parkinson's disease.

The literature on the subject emphasizes that alcohol is the drug most often chosen by older adults, although the frequency of its consumption decreases with age. It should be remembered that the consumption of alcoholic beverages among people of post-working age is hazardous due to the more severe health consequences of drinking in this group than in younger people. High alcohol levels in the body last longer because these individuals experience a decline in muscle mass and increased body fat. Drinking large amounts of alcohol contributes to osteoporosis, liver cirrhosis, atherosclerosis and heart attack, neurological disorders and breast cancer. Also, alcohol consumption is hazardous among people who take medications or suffer from chronic diseases. Unfortunately, chronic, progressive disease, common in seniors, and the inability to accept it, may be an additional cause of addiction (3–5).

Reasons for reaching for alcoholic beverages may also be problems resulting from the aging process, dependence on other people, low social and economic status and loss of a close family member. People with problems of old age start drinking alcohol most often after reaching the age of 65, and the symptoms of intoxication are masked by the environment (5).

Gerstenkorn and Suwała (6) report that half of the seniors had exposure to alcohol in the year prior to their research to varying degrees. Karakiewicz et al. (7) emphasizes that 25% of men over 65 with heart disease drink up to four glasses of vodka a day.

Dabrowska and Wieczorek (8), in the “Report on the implementation of the study: Analysis of risk factors and factors protecting the use of psychoactive substances among seniors,” showed, for example, that the compulsion to drink the next day after drinking alcohol affected 7.5% of their respondents. The percentage of seniors who drank a lot (15%) was comparable to the results of American research. Lehmann and Fingerhood (9) found that 14.5% of Americans aged 65 and over consumed more alcohol than the local “safety” standards (a maximum of three drinks per day and a maximum of seven per week counted in standard alcohol portions).

There are many tools for measuring personality types. One example is the Structure of Temperament Questionnaire (STQ). It was developed by Rusalov within an experimental tradition for studying properties of nervous systems. Using human subjects, Rusalov measured multiple behavioral and psycho-physiological indices, including EEGs and evoked potentials. This resulted in the first activity-specific model of temperament separating physical-motor and social-verbal traits of temperament (10). The Functional Ensemble of Temperament (FET) model was created based on this researcher's tool. It contains links to many neurochemical systems (neurochemistry and neuropharmacology) and uses double-word names of the scales (motor-physical or social-verbal) (10). The concept of affective temperament was also created. This temperament style is characterized by one or more of five main affective dimensions: anxious, irritable, cyclothymic, hyperthymic and depressive (11).

Some authors suggest that one important predictor of treatment outcome, e.g., in depression, is affective experience, specifically the experience of positive affect (PA) and negative affect (NA). PA and NA are two distinct and relatively independent dimensions. They show a moderate correlation between them (12). On the other hand, the five-factor model of personality, created by Costa and McCrae, is a hierarchical organization of personality traits in terms of five basic dimensions: extraversion, agreeableness, conscientiousness, neuroticism and openness to experience (13).

The Temperament and Character Inventory is an objective questionnaire that assesses a seven-factor psychobiologic model of quantifiable personality traits. It has been validated based on various genetic and neurobiological data. Its four temperament dimensions—harm avoidance, novelty-seeking, reward dependence and persistence—are rooted primarily in various neurobiological data. Further, the three character dimensions—self-directedness, cooperativeness and self-transcendence—develop based on social learning (14).

Cox and Klinger believed that personality influences alcohol addiction through specific motives that prompt individuals to drink (15).

However, with regard to people with alcohol addiction, some researches have found the link between personality type D and addiction (16, 17). This stress-prone personality is characterized by negative affect and social inhibition. People with the personality type D experience negative emotions in various situations and inhibit their expression (16). Type D personality is stable across time and has a substantial underlying genetic component (18), and established cross-cultural measurement equivalence (19). The type D personality is a vulnerability factor for future episodes of emotional stress such as depressive episodes, anxiety and addiction to alcohol (16, 17, 20, 21).

In a cross-sectional study, 862 participants (mean age 26.1 years) completed the Type D Personality Scale, Drinking Motives Questionnaire and Severity of Alcohol Dependence Questionnaire; Type D personality was positively correlated with alcohol dependence. Furthermore, this relationship was mediated by coping and conformity drinking motives (16). Williams et al. (17) studied whether Type D personality was associated with higher levels of alcohol use in a group of 138 young participants (mean age 31.8 years). Their results report that Type D was associated with higher levels of alcohol use, stress and desire for alcohol at stressor and recovery.

The present study aimed to assess the style of coping with stress, emotions and anxiety in elder men addicted to alcohol and the relationship between the above. Furthermore, the correlation of Type D personality with alcohol dependence was assessed.

The following research questions were formulated:

- Are people addicted to alcohol satisfied with their lives?
- What level of stress do they feel?
- Do they present Type D personality? People with negative emotionality, social withdrawal, prone to experiencing strong emotions (fear, anger, hostility or irritation), tend to avoid social contact for fear of lack of approval, with a tendency not to manifest their emotions and behavior.
- Is there a statistically significant relationship between the age of the respondents and life satisfaction, having a Type D personality and the stress level presented?

## Materials and Methods

### Study Group

One hundred seventy men aged 60–68 years (mean age - 63 ± 3.1 years), mostly from a rural environment (78%), participated in the study. Twenty-two percentage of respondents lived in the city. The respondents had primary education (23%), vocational (45%), secondary (22%), or higher (10%). The respondents' wealth on a scale of 1–5 points was 3.1 ± 0.2. The above was confirmed by the results of the FAS scale study, where the respondents obtained an average of 3.9 ± 1.9 (min. 1, max. 8), which proves their average level of affluence.

### Study Design

A cross-sectional study was conducted from 2017 to 2020 in patients treated for alcohol addiction in the Alcohol Addiction Therapy Department for Men. Apart from age and 2 weeks abstinence from alcohol before admission to the Department, an additional inclusion criterion was the consent to participate in the study. Mental and physical condition at the admission to the department was assessed. It was satisfactory with no signs of active disease. The exclusion criteria were: (1) age under 60; (2) lack of literacy skills (a respondent who completed at least primary school could have participated in the survey); and (3) refusal to participate in the study. Each participant could withdraw from the study at any time. The completion rate was 100%, as the respondents completed the questionnaires during their stay in the Alcohol Addiction Therapy Department for Men.

The research was conducted with approval of the Local Ethical Committee of the Medical University of Bialystok.

### Research Tools

The study was conducted using a self-questionnaire consisting of a part supplemented by the authors and a part supplemented by the respondent. In addition, Cantril's Ladder (self-anchoring scale) and standardized questionnaires: Family Affluence Scale (FAS), the Satisfaction with Life Scale (SWLS), Perceived Stress Scale (PSS10), and DS-14 scale were used to assess Type D personality.

FAS was used to assess the material resources of the respondent's family (22). Four questions form the basis of FAS: Does your family have a car or a multi-person car (van)? - response categories: no; yes one; yes, two or more; Do you have your room for your use? Response categories: no, yes. How many times in the last 12 months have you taken your family away from home for holidays or holidays? Response categories: I did not travel at all, once, two times, more than two times; and How many computers does your family have? Response categories: none, one, two, more. The FAS scale ranges from 0 to 7 points. For this study, it was divided into four ranges: 0–1 points (extremely poor families); 2–3 points (relatively poor); 4–5 points (average wealth); 6–7 points (wealthy families) (22).

The Satisfaction with Life Scale (SWLS) is five statements participants may agree or disagree with. Instructions are as follows: Using the 1–7 scale below, indicate your agreement with each item by placing the appropriate number on the line preceding that item. Please be open and honest in responding: 7—Strongly agree; 6—Agree; 5—Slightly agree; 4—Neither agree nor disagree; 3—Slightly disagree; 2—Disagree; 1—Strongly disagree (23). The scores obtained were summed up, and the overall result was the participant's degree of satisfaction with his own life. The range of results could be from 5 to 35 points; the higher the score, the greater the sense of satisfaction with life: 31–35 points—extremely satisfied; 26–30 points—satisfied; 21–25 points—slightly satisfied; 20 points—neutral; 15–19 points—slightly dissatisfied; 10–14 points—dissatisfied; 5–9 points—extremely dissatisfied. In the interpretation of the result, the properties characterizing the sten scale were also applied. Results in the ranges 1–4 of the sten were treated as low; 7–10 as high; and 5 and 6 as average. The reliability index (Cronbach's alpha) of SWLS is 0.81. The scale stability index, determined in the double study of 30 people with an interval of 6 weeks, was 0.86 (23).

Cantril's Ladder (self-anchoring scale) has a graphical form of a ladder, with steps marked with consecutive numbers from 0 to 10. Next to the ladder, a text explains that the top (10) is the best life there can be, and the bottom (0) is the worst life (24, 25). The respondent is to decide what his life is like at the moment and put an X in the appropriate place on the ladder. It is assumed that 0–5 points are obtained by people dissatisfied with their life; 6–8 points—average satisfaction; 9–10 points—very satisfied (24, 25).

The PSS10 contains 10 questions about various subjective feelings related to personal problems and events, behaviors and ways of coping (23). The respondents answered by entering the correct number (0—never, 1—rarely, 2—sometimes, 3—quite often, 4—very usually). The overall score is the sum of all points, with a theoretical distribution from 0 to 40. The higher the score, the greater the severity of the perceived stress. The overall index after conversion to standardized units is interpreted according to the properties characterizing the sten scale. Scores in the range 1–4 sten are treated as low, 7–10 sten as high and 5–6 sten as average (23).

The DS-14 scale to assess Type D personality *-* an adaptation by Ogińska-Bulik et al. (26) of the Type D Personality Scale (DS14) by Johan Denollet (27), was used for the measurement of distressed personality. The scale includes 14 statements; seven refer to negative emotionality (NE) and seven to social inhibition (HS). The examined person responds to the given statements using a five-point scale (0—false to 4—true). To qualify for Type D, the respondent must obtain at least 10 points in both dimensions. The opposite of Type D is non-D (both sizes result below 10 points).

### Statistical Analysis

Statistical analysis of data was conducted using Statistica® version 13.3. We used descriptive statistics such as percentage calculation of results, arithmetic mean, standard deviation, tabular and graphical representation of data.

## Results

The study included 170 men treated in the Alcohol Addiction Therapy Department for Men. The longest duration of alcohol consumption was 35 years, the shortest-15 years (24.3 ± 14.2 years). The therapy lasted from 1 to 7 weeks (3.5 ± 2.1 weeks).

The subjects used Cantril's Ladder to decide what their life was like at the examination time. The respondents obtained an average of 5.5 ± 1.9 points. The respondents most often marked 5 points (47.1%) and least frequently marked 9.8, and 4 (5.6%). The results obtained indicate that the respondents were, on average satisfied with their lives.

According to the SWLS scale, the respondents were very dissatisfied with their lives (17.2 ± 4.9). Detailed results are presented in Table 1. In the group dissatisfied with their lives, 5.9%, very dissatisfied with their lives−17.6%, rather dissatisfied with their lives−41.2%, neither satisfied nor dissatisfied with their lives−5.9% and somewhat satisfied with their lives−29.4%.

**Table 1:** Assessment of life satisfaction of the respondents assessed using the SWLS scale.

	**Strongly agree**	**Agree**	**Slightly agree**	**Neither agree nor disagree**	**Slightly disagree**	**Disagree**	**Strongly disagree**
In most ways, my life is close to my ideal			11.8%	11.8%	41.2%	11.8%	23.4%
The conditions of my life are excellent		17.6%	29.4%	17.6%	17.6%	11.9%	5.9%
I am satisfied with my life		11.8%	29.4%	17.6%	17.6%	11.8%	11.8%
So far I have gotten the important things I want in life		5.9%	17.6%	41.2%	29.4%	5.9%	
If I could live my life over. I would change almost nothing			17.6%	11.8%	17.6%	29.4%	23.6%

There was no significant correlation between the age of the respondents and life satisfaction (*p* = 0.36339), having a Type D personality, age (*p* = 0.20274) and wealth (*p* = 1.000). Significant correlations between age and stress level (*p* = 0.00524) and between affluence and life satisfaction (*p* = 0.00077) and level of stress (*p* < 0.001) were found. Detailed results are presented in Tables 2, 3.

**Table 2:** Assessment of the perceived stress using the PSS-10 scale.

	**Never**	**Almost never**	**Sometimes**	**Fairly often**	**Very often**
In the last month, how often have you been upset because of something that happened unexpectedly?			58.8%	23.5%	17.7%
In the last month. how often have you felt that you were unable to control the important things in your life?		23.5%	17.6%	41.1%	17.8%
In the last month. how often have you felt nervous and “stressed”?		10.9%	17.1%	47.1%	24.9%
In the last month. how often have you felt confident about your ability to handle your personal problems?	33.5%	17.6%	24.7%	24.2%	
In the last month. how often have you felt that things were going your way?	16.8%	24.4%	23.5%	35.3%	
In the last month. how often have you found that you could not cope with all the things that you had to do?	5.9%	10.9%	24.4%	41.2%	17.6%
In the last month. how often have you been able to control irritations in your life?	5.9%	17.6%	35.3%	41.2%	
In the last month. how often have you felt that you were on top of things?	17.7%	17.6%	52.9%	5.9%	5.9%
In the last month. how often have you been angered because of things that were outside of your control?		11.8%	24.9%	46.2%	17.1%
In the last month. how often have you felt difficulties were piling up so high that you could not overcome them?	5.8%	5.9%	41.2%	35.3%	11.8%

**Table 3:** Type D personality in the study group.

	**False**	**Rather false**	**Neutral**	**Rather true**	**True**
I make contact easily when I meet people		11.8%	23.5%	41.2%	23.5%
I often make a fuss about unimportant things		11.8%	23.5%	24.9%	39.8%
I often feel unhappy	5.9%	23.5%	17.6%	24.9%	28.1%
I am often irritated	5.9%	17.6%	23.5%	35.3%	17.7%
I often feel inhibited in social interactions		17.6%	23.5%	41.3%	17.6%
I take a gloomy view of things	5.9%	11.8%	23.5%	35.3%	23.5%
I find it hard to start a conversation	5.9%	24.9%	24.9%	24.9%	19.4%
I am often in a bad mood	5.9%	11.8%	24.9%	24.9%	32.5%
I am a closed kind of person	5.9%	35.3%	24.9%	17.6%	16.3%
I would rather keep other people at a distance	5.9%	17.6%	5.9%	58.8%	11.8%
I often find myself worrying about something	5.9%	23.5%	5.9%	41.2%	23.5%
I am often down in the dumps		35.3%	17.6%	24.9%	22.2%
When socializing. I don't find the right things to talk about above		35.3%	23.5%	20.4%	20.8%

## Discussion

The study aimed to assess the style of coping with stress, emotions, and anxiety, Type D personality in elder men addicted to alcohol, and the relationship between the above.

The participants were very dissatisfied with their lives, with a high level of perceived stress and Type D personality. There was no significant relationship between the respondents' age and life satisfaction or respondents' age, having a Type D personality, and wealth. However, there was a correlation between age and level of stress and wealth, life satisfaction and stress level.

It is estimated that 2.3 billion people worldwide drink alcohol today. In terms of average consumption per capita, Europe is the leader, even though alcohol consumption has fallen by more than 10% since 2010. According to WHO, global alcohol consumption will increase in the next 10 years, especially in Southeast Asia, the Western Pacific and the Americas. Europeans and Americans drink the most alcohol, averaging 9.8 and 8 L of pure ethyl alcohol per day, respectively (28). In 2019, among men, respondents aged 45–54 used alcohol most often; in this group, it is practically common at 99%, and 83% in people over 65. Vodka was used by 22% of people aged 55–64 and 26% of people over 65. Seventy-three percentage of people over 65 used alcohol more often with their family than with friends (29).

It is worth emphasizing that alcohol abuse among seniors does not always have to refer to the stereotypical consumption of alcohol during loud feasts, and sometimes it may refer to drinking one's favorite beer before bedtime or a large glass of wine every evening (28, 29). The problem begins when alcohol becomes a habit, and a person cannot imagine a day without it. Unfortunately, in recent times, the context of alcohol use and aging is a common phenomenon, both among men and women. Such alcohol consumption causes irreversible changes and damage to internal organs and consequently, reduces the life expectancy and condition of the senior's body drastically. Excessive alcohol consumption causes individuals to neglect daily duties, including, for example, missing regular visits to the doctor and not carrying out tests. Addicts often ignore the disease symptoms and do not care about their health until it is, unfortunately, too late to start treatment. Furthermore, hyperkatifeia is very interesting fenomenon. It is defined as the negative emotional state of drug withdrawal. It drives the negative reinforcement source of motivation for compulsive-like alcohol seeking and using. Moreover, hyperkatifeia can drive impulsivity like relapse in the preoccupation or anticipation stage and then a return to the binge or intoxication stage (30).

Data from the UK (31) summarizing hospital admissions related to alcohol abuse indicate that among the 337,110 people admitted, 56,240 were aged 64–75, and 43,910 were over 75 years of age. Thus, the cited data confirm a relatively large percentage of seniors in this group.

In a Polish study (5) that included 110 older adults living in Lubelskie Voivodeship, Poland, most seniors (83.6%) used alcohol occasionally. In this study, alcohol consumption was more common among older adults from rural rather than urban environments, and the majority of alcohol drinkers had primary education and were single. Indeed, the low level of education implies minor criticism of the older adults in relation to the advice given to them by other people. Also, in the present study, the majority of respondents came from the country.

In the study conducted by Wadd and Papadopoulos (32), older adults experienced high levels of alcohol-related harm but drink less than younger adults, and levels of alcohol-related harm in older adults are increasing. Similar findings were described in a US study (33), where single people (without support from family members, divorced, widowed) were more likely to seek consolation through drinking alcohol.

Researchers studying with alcohol addiction have long been looking for factors that may play an essential role in the genesis of addiction and the course of treatment (34). Only a small number articles have been published on the life satisfaction and quality of life of alcohol addicts.

In the analysis of life satisfaction of the elderly population in the example of Sweden, Austria and Germany, the author found that aging does not necessarily worsen one's perception of life. Based on the data shown here, there is no evidence from Austria and Spain that all people systematically, regardless of the year of birth, go through a stage of a lower level of life satisfaction. An important factor of life satisfaction is health self-assessment (35).

Data from the literature indicate the coexistence of anxiety, depressive disorders, somatization and phobias to a greater extent in women addicted to alcohol than in men. Also, the role of fear, optimism, self-efficacy and life satisfaction was studied (34). People also see alcohol as a way to cope with economic stress, stress at work and marital problems, often due to a lack of social support (36). Whether an individual will drink under stress depends on many factors, including genetics, the individual's drinking behavior, type of stressor, personal sense of control stress and the range of stress coping methods (36–39).

Some researchers believe that high levels of stress impact alcohol consumption when there are no alternative resources; when alcohol is available, the person believes alcohol will help reduce stress (36–39). This is confirmed by the current study, which shows that those respondents had a high level of perceived stress.

However, according to Pohorecky (36), whether people drink under stress beyond their control is not obvious. In his review of research on the relationship between alcohol consumption and stress, Pohorecky took into account several studies that examined people living in areas affected by a natural disaster. One study found a 30% increase in alcohol consumption in the 2 years following the Buffalo Creek floods in West Virginia. Similar increases were recorded in cities near Mount St. Helens after a volcanic eruption (35). However, after the Three Mile Island nuclear power plant disaster, alcohol was rarely used to deal with stress (39). Some studies show that drinking can occur when stress is anticipated or experienced (40).

It is also worth remembering that, in non-drinking people addicted to alcohol, personally threatening, severe and chronic life stressors can lead to alcohol relapse (37, 41). Brown (41) conducted a study on a group of men who discontinued inpatient therapy and then experienced acute and chronic psychosocial stress. The researchers found that patients who relapsed experienced acute and chronic stress twice as often before returning to drinking as those who continued abstinence. The authors believe that while many factors contribute to the resumption of drinking, stress may have the most significant impact on the first alcohol consumption after a period of abstinence (42).

Research on the personality types of people addicted to alcohol and attempts to create a uniform personality profile has not yielded unequivocal results. Therefore, Cloninger et al. (43) investigated the inheritance of personality traits in susceptibility to alcohol addiction. In their prospective, longitudinal study, three dimensions of personality that could be a harbinger of a later addiction were distinguished: seeking novelty and impressions, avoiding unpleasantness and dependence of behavior on reward, but these features were not correlated with each other.

It is well-known that certain personality traits are associated with alcohol use (44). Because less is known about these traits, we wished to investigate whether changes in alcohol use were longitudinally associated with personality changes and in which direction the influence or causation might flow. Data came from the self-reported questionnaire answers of 5,125 young men at two time points during the Cohort Study on Substance Use Risk Factors (C-SURF). Their average ages were 20.0 and 25.4 years old at the first and second wave assessments, respectively. Four personality traits were measured: (a) aggression–hostility; (b) sociability; (c) neuroticism-anxiety; and (d) sensation-seeking. Alcohol use was measured by volume (drinks per week) and binge drinking (about 60+ g per occasion). Cross-lagged panel models and two-wave latent change score models were used. Aggression-hostility, sensation-seeking and sociability were significantly and positively cross-sectionally associated with both alcohol use variables. Drinking volume and these three personality traits bidirectionally predicted each other. Binge drinking was associated with sensation-seeking only, whereas aggression-hostility and sociability only predicted binge drinking, but not vice versa. Changes in alcohol use were significantly positively related to differences in aggression-hostility, sensation-seeking and sociability. Associations reached small Cohen's effect sizes for sociability and sensation-seeking, but not for aggression-hostility. Associations with neuroticism-anxiety were mainly not significant. The direction of effects confirmed findings from other studies, and the association between changes in personality and alcohol use supports the idea that prevention programs should simultaneously target both.

In the present study, we tried to assess whether the study participants addicted to alcohol belong to Type D personality, i.e., tend to feel negative emotions (negative emotionality), e.g., anger, fear, have a pessimistic approach to life, constantly feel tension, worry, feel insecure, are withdrawn in social relationships (social inhibition), and are reluctant to show negative emotions due to fear of rejection.

Our current study has some potential limitations. First, the study group was too small to generalize the results to Poland's entire population of drinking seniors. The study concerned only men, so it is worth verifying the results in other studies in an equally large group of women. It is known that alcohol consumption among women differs from that among men. Women have different prognostic factors for alcohol abuse than men. Intoxication occurs in women after drinking less alcohol; chronic alcohol abuse causes more deaths in women; and alcohol has a more harmful effect on women's health than in men.

Despite these limitations, the results of this study may constitute a starting point for further research into the problems of older people addicted to alcohol. Optimally, these findings should be verified in a longitudinal examination and extended to a control group.

## Conclusions

The men surveyed were very dissatisfied with their lives, had a high level of perceived stress and displayed personality traits of Type D. No significant relationships were found between the age of the respondents and life satisfaction and their wealth and having a Type D personality. Significant associations between age and the level of stress and affluence and the level of stress and life satisfaction were found.

## Data Availability Statement

The original contributions presented in the study are included in the article/supplementary material, further inquiries can be directed to the corresponding author.

## Ethics Statement

The studies involving human participants were reviewed and approved by the Local Ethical Committee of the Medical University of Bialystok. The patients/participants provided their written informed consent to participate in this study.

## Author Contributions

GB created the research concept. AK-B and GB were collecting materials to the study and prepared the initial version of the manuscript, which was corrected, supervised, and completed by NW and EK-K. EK-K conducted data analysis. All authors contributed to the article and approved the submitted version.

## Conflict of Interest

The authors declare that the research was conducted in the absence of any commercial or financial relationships that could be construed as a potential conflict of interest.

## Publisher's Note

All claims expressed in this article are solely those of the authors and do not necessarily represent those of their affiliated organizations, or those of the publisher, the editors and the reviewers. Any product that may be evaluated in this article, or claim that may be made by its manufacturer, is not guaranteed or endorsed by the publisher.
